# Anesthesiologist Provided Regional Nerve Block Against Surgeon Provided Infiltration Block for Abdominal Surgery: Case Series

**DOI:** 10.7759/cureus.19606

**Published:** 2021-11-15

**Authors:** Poonam Pai BH, Samiat Jinadu

**Affiliations:** 1 Anesthesiology, Mount Sinai Hospital, New York City, USA; 2 Anesthesiology, Oregon Health & Science University, Portland, USA

**Keywords:** mastectomy, breast reconstruction, regional nerve block, american society of anesthesiologists (asa), transversus abdominis plane (tap)

## Abstract

We present two patients who underwent double mastectomy and breast reconstruction with deep inferior epigastric artery perforator (DIEP) flap. The goal of this case series was to compare surgeon-provided infiltration block against anesthesiologist-provided regional nerve block, focusing on abdominal analgesia. This case report highlights that pain control for a patient could be successful when done collaboratively. To achieve this, it is important for both the surgical and anesthesia team to discuss the best analgesic plan for the patient while taking into consideration the confidence, experience, and technique that both the surgical and anesthesiology team can offer.

## Introduction

Post-mastectomy breast reconstruction is an extensive surgery that poses interesting challenges for post-operative pain control as the breasts and donor site (usually, the abdomen) must both be taken into consideration. Multimodal analgesia is the framework employed to ensure adequate analgesia while minimizing medication side effects and opioid burden. Surgical infiltration of local anesthetic into the abdominal incision has been used to provide analgesia. However, with the increasing popularity of regional anesthetic nerve blocks, such as the ultrasound-guided transversus abdominis plane (TAP) block, there is often a discussion regarding which technique would be more beneficial for patients. Keeping this background in mind, we investigated this question by comparing surgeon-provided infiltration block against anesthesiologist-provided regional nerve blocks focusing on abdominal analgesia.

Both patients provided Health Insurance Portability and Accountability Act (HIPAA) authorization for the publication of this report.

## Case presentation

**Figure 1:**
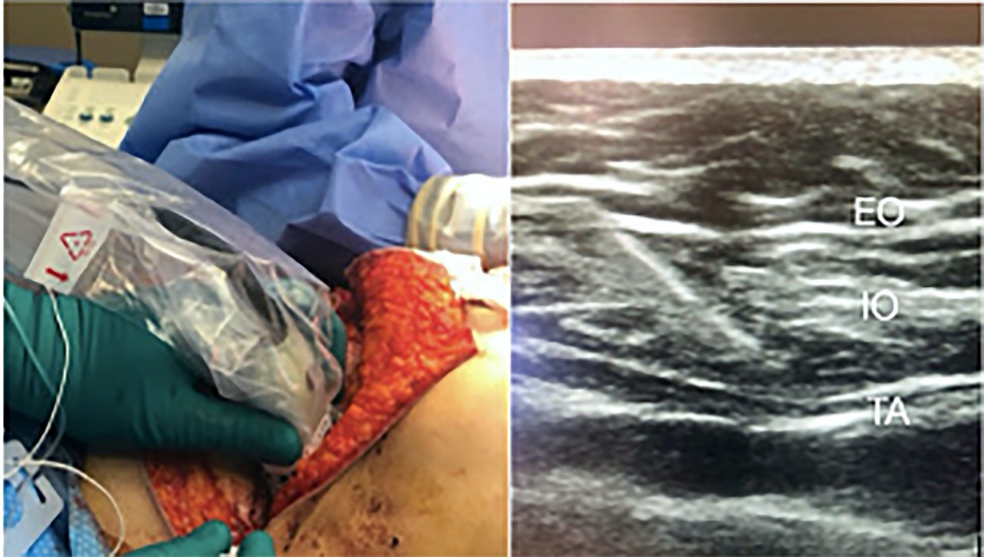
TAP block performed by the surgical team on the left side with an ultrasound. On the right is the sonographic image with the local anesthetic being deposited between the internal oblique (IO) and transversus abdominis (TA) muscle in a TAP block performed by an anesthesiologist. EO = external oblique muscle, TAP = transversus abdominis plane.

We present two patients who underwent double mastectomy and breast reconstruction with deep inferior epigastric artery perforator (DIEP) flap for breast cancer. After placement of standard American Society of Anesthesiologists (ASA) monitors, general anesthesia was induced, and patients were intubated. The surgical team proceeded with bilateral mastectomy. Subsequently, an inferior lower abdominal incision was made for the harvesting of the DIEP abdominal flap. Prior to the closure of the flap, the surgical team, with a sterile linear ultrasound probe on the agreed-upon side of the abdomen, injected 20ml of a mixture of 10ml liposomal bupivacaine and 10ml 0.25% bupivacaine into the fascial layer between the internal oblique and transversus abdominis (Figure 1). The procedure was successfully completed. After which, prior to extubation, the anesthesia team placed a unilateral ultrasound-guided TAP block, on the alternate side, with the same mixture of local anesthetic as mentioned above. A high-frequency linear array (5-13 MHz) (Fujifilm Sonosite Inc., Bothell, WA) probe and a 50 mm echogenic needle (B. Braun Medical Inc., Bethlehem, PA) was used for both the blocks. Both patients had uneventful surgeries. They were extubated and transferred to the recovery unit. After which, pain scores at rest and dermatomal distribution of the block, using pin-prick test were assessed at 0, 6, 12, and 24 hours. The results are described in Tables 1, 2. Both patients had a hospital length of stay of three days. The duration of surgery for patient A and patient B was 5.39 hours and 8.22 hours, respectively.

**Table 1:** Pain scores at rest and dermatomal distribution for patient A

Time	Pain Scores at rest		Dermatomes	
	Surgeon-provided TAP	Anesthesiologist-performed TAP	Surgeon-provided TAP	Anesthesiologist-performed TAP
Q0	2	0	T8-T10	T8-L1
Q6	0	0	T7-T9	T7-T9
Q12	5	5	No dermatomal coverage	No dermatomal coverage
Q24	0	0	No dermatomal coverage	No dermatomal coverage

**Table 2:** Pain scores at rest and dermatomal distribution for patient B

Time	Pain Scores at rest		Dermatomes	
	Surgeon-provided TAP	Anesthesiologist-performed TAP	Surgeon-provided TAP	Anesthesiologist-performed TAP
Q0	5	5	No dermatomal coverage	T11-T12
Q6	0	0	No dermatomal coverage	T11-T12
Q12	1	1	No dermatomal coverage	T11-T12
Q24	5	5	No dermatomal coverage	No dermatomal coverage

Case A

Patient A was a 62-year-old female with a medical history of hypothyroidism, hypertension with a BMI of 23kg/m^2^. Patient A received 17mg morphine milligram equivalents in the first 24 hours. Patient A had adequate, comparable pain control with both the surgeon and anesthesiologist provided TAP blocks.

Case B

Patient B was a 42-year-old female with a history of thyroid cancer with a BMI of 27kg/m^2^. Patient B received 45mg morphine milligram equivalents in the first 24 hours. Patient B reported moderate pain bilaterally, although there was a discernable dermatomal coverage on the side with the anesthesiologist-provided TAP block.

## Discussion

During the initial evaluation, our team was confident that an ultrasound-guided TAP block would be more beneficial for the patient than a surgical infiltration. However, the surgeon was also confident that they could provide better analgesia with a variation of the ultrasound-guided TAP block. We did not see any studies comparing the efficacy and theoretical safety profile of surgeons performing TAP block to conventional ultrasound-guided TAP block, and this prompted this investigation to compare both. The eventual comparable pain control on both sides of the abdomen can be explained by the concurrent use of ultrasonography by both teams.

The first TAP block was described as an anatomic landmark guided field block for abdominal surgeries [1]. The nerves which innervate the anterior abdominal wall travel between the internal oblique and transverse abdominis. Since its original description as a series of “fascial clicks” in the lumbar triangle of petit, the TAP block has been used extensively for abdominal surgeries with the evolution of different variations. The inside-out method was first described by Owen et al., who performed the block intra-abdominally without the use of ultrasound; this method was proposed as an alternative to conventional ultrasound-guided TAP block, which carries the potential risk of peritoneal injury [2].

The advent of ultrasound has made the TAP block a safe procedure while ensuring the correct placement of the local anesthetic into the correct plane [3]. Hebbard et al. described an ultrasound-guided TAP block where the fascial planes were identified accurately to deposit local anesthetic between the internal oblique and transversus abdominis [4]. Nash et al. described a surgical technique of performing a TAP block where a sterile probe was placed directly onto the exposed external oblique muscle [5]. The removal of the skin and subcutaneous fat layers allowed for better ultrasound demonstration of the muscle layers and a shallower needle angle [5].

A study looking at surgeon-administered intraoperative TAP block in abdominal reconstruction surgery concluded that the patients, when compared to no block, had a shorter hospital length of stay, lower opioid consumption, and reduced nausea and vomiting [6]. The use of a surgeon-administered TAP block was argued to be advantageous because the surgeons could engage in concurrent activities such as drain placement and flap insertion during the placement of the block, therefore, not adding any appreciable time to the procedure. However, an anesthesiologist-performed TAP block in the hands of a skilled provider can be argued not to add significant operating time.

Conventional ultrasound TAP blocks have been shown to have analgesic efficacy in several randomized controlled trials (RCTs) with a reduction of opioid consumption and pain scores. A study by Momeni et al. showed that 91.3% of patients who received liposomal bupivacaine for intra-abdominally placed blind surgical TAP block did not require patient-controlled analgesia (PCA) postoperatively [7]. A Cochrane review concluded that there is some evidence to suggest TAP blocks decrease opioid consumption and pain scores when compared with placebo [8].

## Conclusions

A randomized controlled trial comparing an anesthesiologist performing TAP to a surgeon performing TAP may shed further light on analgesic efficacy between them. These cases highlighted that pain control for patients could be successful when done collaboratively. To achieve this, it is important for both the surgical and anesthesia team to discuss the best analgesic plan for the patient while considering the confidence, experience, and technique that both the surgical team and anesthesiology team can offer.

## References

[1] Rafi AN (2001). Abdominal field block: a new approach via the lumbar triangle. Anaesthesia.

[2] Owen DJ, Harrod I, Ford J, Luckas M, Gudimetla V (2011). The surgical transversus abdominis plane block—a novel approach for performing an established technique. BJOG.

[3] Kadam RV, Field JB (2011). Ultrasound-guided continuous transverse abdominis plane block for abdominal surgery. J Anaesthesiol Clin Pharmacol.

[4] Hebbard P, Fujiwara Y, Shibata Y, Royse C (2007). Ultrasound-guided transversus abdominis plane (TAP) block. Anaesth Intensive Care.

[5] Nash H, Khoda B, Heppell S, Turner M (2011). TAP blocks in breast reconstructions using abdominal wall tissue. Anaesthesia.

[6] Wheble GA, Tan EK, Turner M, Durrant CA, Heppell S (2013). Surgeon-administered, intra-operative transversus abdominis plane block in autologous breast reconstruction: a UK hospital experience. J Plast Reconstr Aesthet Surg.

[7] Momeni A, Ramesh NK, Wan D, Nguyen D, Sorice SC (2019). Postoperative analgesia after microsurgical breast reconstruction using liposomal bupivacaine (Exparel). Breast J.

[8] Charlton S, Cyna AM, Middleton P, Griffiths JD (2010). Perioperative transversus abdominis plane (TAP) blocks for analgesia after abdominal surgery. Cochrane Database Syst Rev.

